# Trends in the consumption rates of benzodiazepines and benzodiazepine-related drugs in the health region of Lleida from 2002 to 2015

**DOI:** 10.1186/s12889-020-08984-z

**Published:** 2020-06-01

**Authors:** F. Torres-Bondia, J. de Batlle, L. Galván, M. Buti, F. Barbé, G. Piñol-Ripoll

**Affiliations:** 1 Pharmacy Department, Clinical Neuroscience Research, IRBLleida, Arnau de Vilanova University Hospital, Lleida, Spain; 2 Biomedical Research Networking Center in Respiratory Diseases (Centro de Investigación Biomédica en Red de Enfermedades Respiratorias, CIBERES), Madrid, Spain; 3 Group of Translational Research in Respiratory Medicine, Arnau de Vilanova University Hospital and Santa Maria University Hospital, IRBLleida, Lleida, Spain; 4 Pharmacy Department, Servei Català de la Salut (Catalan Health Services), Lleida, Spain; 5 Unitat d’Avaluació Clínica (Clinical Evaluation Unit), Institut Català de la Salut (Catalan Institute of Health), Lleida, Spain; 6 Unitat Trastorns Cognitius (Cognitive Disorders Unit), Clinical Neuroscience Research, IRBLleida, Santa Maria University Hospital, Rovira Roure n° 44, 25198 Lleida, Spain

**Keywords:** Benzodiazepines, Sedative-hypnotics, Prescribing trends, Drug safety, Drug utilization

## Abstract

**Background:**

The high prevalence and long-term use of benzodiazepines (BZDs) treatment are debated topics because of the risk they can cause to the patients. Despite the current information on the risk-benefit balance of these drugs, their consumption remains particularly high. We determined the trend in the consumption prevalence of benzodiazepines (BZDs) and drugs related to BZDs (Z-drugs) in the population of the Health Region of Lleida to explore patterns of use and the associated characteristics associated between 2002 and 2015.

**Methods:**

An analysis of secular trends was carried out between 2002 and 2015; the databased included all individuals from the Health Region of Lleida, which had 358,157 inhabitants in 2015, that consumed BZDs. The consumption of BZDs was evaluated using prescription billing data from the Public Health System. All types of BZDs and BZD analogues that had been approved by the drug agency were included. Trends by age and sex were investigated.

**Results:**

Over the whole study period, a total of 161,125 individuals accounted for 338,148 dispensations. Overall, 59% were women, and the mean age was 56 years. The dispensing prevalence of BZDs use in 2015 was 14.2% overall —18.8% in women and 9.6% in men—and was 36% in those over 65 years. According to the half-life of BZDs, the prevalence of short-intermediate BZD use, intermediate-long BZD use, and Z-drugs use was 9.7, 5.5 and 0.8%, respectively. The evolution of the annual prevalence of BZD dispensing showed a progressive decline, from 15.3% in 2002 to 14.2% in 2015, which was attributed to a decrease in the consumption of intermediate-long half-life BZDs (8.0% vs. 5.5%) and Z-drugs (1.4% vs. 0.8%).

**Conclusion:**

The dispensing prevalence of BZDs and Z-drugs was high, although a small reduction was observed during this time period. The dispensing prevalence was especially high in the population over 65, despite the risk of cognitive decline and falls. Integral actions are required to lower the BZD prescription rate.

## Background

Benzodiazepines (BZDs) and drugs related to benzodiazepines (Z-drugs) are one of the most highly used pharmacological groups globally, especially in developed countries [1, 2]. Their consumption has reached 30% for people over 65 years of age in France [3], more than 20% in Canada [4] and Spain [5], 15% in Australia [6], and between 9 and 12% in the US [7, 8].

These drugs are mainly indicated for the treatment of generalized anxiety disorder and insomnia, although they are also indicated for panic attacks, phobic disorders, obsessive-compulsive disorders, post-traumatic stress, epilepsy, and muscle spasms [9]. In adults older than 65 years, good practice guidelines do not recommend their use in the treatment of insomnia, agitation, or delirium and it is recommended that BZD use, if any, be short-term [10]. Thus, a maximum duration of between 2 and 4 weeks for insomnia or anxiety and no more than 2 weeks for mixed anxiety-depressive disorders are advised [11]. These recommendations were based on the long-term development of tolerance, dependence, abuse and withdrawal syndrome [12], although recent retrospective studies showed a level of dependence after chronic consumption that was lower than previously described, even in older people [13]. In any case, BZDs have been demonstrated to increase the risk of falls, hip fractures and detrimental cognitive effects [14, 15].

Despite these recommendations, the use of BDZs is a cause for concern in the public health systems of different countries due to their high prescription prevalence and the risks associated with long-term exposure [16].

Most BZDs are prescribed by primary care services due to the greater number of elderly patients who visit these practices [7]. However, in relative terms, psychiatrists present the highest prescription rates [17]. According to previous studies, only 20–30% of prescription of BZDs prescriptions are appropriate [18, 19]; in Canada, this means an extra cost of CAN $3076 per person per year in hospitalization, emergency department and outpatient costs [20]. In older people, two aspects as hospitalization and nursing home admission, are triggers for the initiation of BZDs; between 13 and 69% of hospitalized older people use BZDs [21, 22], and the prevalence of BZD use in nursing home residents is 25–50% [23–25].

The chronic use of BZDs has a great impact on the elderly beyond the risk of dementia, traffic accidents or falls. Thus, other aspects such as a fear of falling, tripping, almost falling, loneliness, social isolation, depression or reduced of social participation can be side effects of treatment with BZDs [26]. Thus, people with a higher risk of falls have lower functional activity and social participation, which can lead to a higher risk of depression and anxiety, an increased use of BZD and, subsequently, more falls [27]. All these aspects also have economic and social consequences; thus, the estimated additional costs of the falls associated with BZD in the European Union were 1800 million euros per year [28].

The general objective of this study was to determine the dispensing prevalence and characteristics of BZD and Z-Drug consumption in a Spanish cohort over a period of 14 years. The specific objectives were to explore the dispensing prevalence of BZD use, the types of BZDs (short-intermediate half-life or intermediate-long half-life) and Z-drugs prescribed, the age and sex characteristics associated with long-term consumption, and the use trends during this time period.

## Materials and methods

This was an analyses of secular trends carried out between January 1, 2002 and December 31, 2015. The database consisted of all individuals of any age and sex who were assigned, either by a doctor or by the Basic Health Area (the Basic Health Area corresponds to the territory and its population, which is attended by a Primary Care Team, whose basic nucleus is made up of professionals from Family Medicine, Paediatrics, Nursing and administrative support staff), to the Health Region of Lleida (Catalonia), with had a catchment area of 358,157 inhabitants in 2015.

To assess the consumption of BZDs and Z-drugs, pharmacy dispensing information from the Public Health System was used; this information contains the number of packages dispensed in the pharmaceutical offices under their charge. Spain has a public health system where the drugs are dispensed in pharmaceutical offices after showing a prescription by a doctor (usually prescribed by a general practitioner or sometimes by specialist in ambulatory patients). This study contains dispensing information from pharmaceutical offices; the following assumptions: consumption associated with mutual insurance entities or other insurers, drugs administered to hospitalized patients, drugs prescribed by private providers who dispensed drugs without a prescription were excluded. In Spain, these combined avenues represented less than 2% of drug consumption.

The best source of data for studies to evaluate the prescription and consumption of drugs, are pharmacy dispensing records because they are based on actual purchases of drugs. Both the external and internal validity of these studies are high. Therefore, the current dispensing records make it possible to carry out a highly reliable analysis at the individual level of drug consumption [29, 30].

It has been observed that patients who consume BZDs continue their prescriptions over time, and the data suggest that BZD use is more likely to be underestimated than overestimated [31]. It can be assumed, therefore, that the purchased treatments were in fact consumed [31]. Therefore, the use of this source of information allowed a good approximation of actual consumption; however, depending on the prevalence of acute or occasional uses, an overestimation is possible when using dispensing data. Anxiolytic and hypnotic BZDs were defined following the anatomical therapeutic classification (ATC) of the World Health Organization (WHO) [32]: N05BA (anxiolytics, BZD derivatives), N05CD (hypnotics and sedatives, BZD derivatives), and N05CF (hypnotics and sedatives, benzodiazepine-related drugs, also called Z-drugs).

All BZDs and Z-drugs in the aforementioned groups, which were approved in the drug catalogue of the Spanish Agency for Medications [33], were included during the study period. Zaleplon was excluded because it was not marketed in Spain during the study period.

The consumption of specialized pharmaceuticals was expressed as the defined daily dose (DDD). The DDD is a technical unit of measurement that corresponds to the daily maintenance dose of a drug for its main indication in adults and for a given route of administration. The DDDs of active ingredients are established by the WHO and are published on the WHO Collaborating Center for Drug Statistics Methodology website [32].

Long-term consumption over the whole study period was defined as a DDD ≥ 180 DDD [34].

The following clinical and demographic variables were recorded: age; sex; type of Basic Health Area (classified as rural or urban) [35]; diagnoses (hypertension, diabetes mellitus, hyperlipidaemia, myocardial infarction, stroke, Alzheimer’s disease or other dementia, anxiety, insomnia and depressive syndromes) according to the International Classification of Diseases, 10th revision (2018), Clinical Modification (ICD-10-CM) [36]; type of BZD, classified according to half-life, (short-intermediate or intermediate-long); and total number of BZDs.

A description of the study population was created based on absolute values and percentages or means and standard deviations. To calculate the percentages of the total population of the Health Region of Lleida, the official figures for that region were used. The dispensing prevalence of BZDs use was calculated by age, sex, and type of BZD (according to half-life) for individuals of any age who were charged for at least 1 prescription for any selected drug between January 1, 2002, and December 31, 2015. We considered global dispensing prevalence when we described the whole study period and annual dispensing prevalence when we described use over a given year.

This research project, with code P16/109, was approved by the reference ethics committee (Committee of Ethics and Clinical Research of Lleida - CEIC).

## Results

During the period 2002–2015, the cohort of BZD and/or Z-drug consumers during the period 2002–2015 in the Health Region of Lleida consisted of 161,125 subjects. These individuals generated 338,148 records of dispensed treatments, which included all possible BZDs and Z-drugs. Table 1 shows the overall characteristics of the study population.

**Table 1:** Characteristics of the cohort of the study population between 2002 and 2015

	n (%)
Sex	
Men	66,685 (41%)
Women	94,440 (59%)
Age categories	
< 16	1293 (1%)
16–24	3676 (2%)
25–44	36,165 (23%)
45–64	48,943 (30%)
> 64	71,048 (44%)
Setting: rural	96,145 (60%)
Main diagnoses	
Alzheimer’s	1172 (0.7%)
Dementia	3710 (2.3%)
Depression	18,931 (11.8%)
Anxiety	37,933 (23.5%)
Sleep disorders	3041 (1.9%)
Affective disorders	4159 (2.6%)
IC	7228 (4.5%)
HTA	48,619 (30.2%)
Diabetes	19,774 (12.27%)
Dyslipidaemia	38,976 (24.2%)
Other	130,786 (81.2%)

*IC* Ischemic cardiomyopathy, *HTA* Hypertension

In the last year of follow-up (2015), the mean (SD) age was 56 (24) years. A total of 59% of the consumers were women, and 60% of the total cohort was assigned to the Basic Rural Area. In our study, the most frequent diagnoses associated with BZDs were anxiety (24%) and depression (19%), while insomnia accounted for only 2% of the recorded diagnoses. In this same year, 50,725 people were prescribed at least one BZD, representing an annual dispensing prevalence of 14.2%, which was higher in women (18.8%) than in men (9.6%). This use increased with age, reaching 36.1% in people over 65 years of age (Table 2). According to the type of BZD (Table 2), the annual dispensing prevalence was 9.7% for the short-intermediate half-life BZDs and 5.5% for the intermediate-long half-life BZDs. In the case of Z-drugs, the dispensing prevalence was 0.8%.

**Table 2:** BZD and Z-drugs consumption dispensing prevalence in 2015 (%)

	< 16	16–24	25–44	45–64	≥65	Total
**Men**						
Short-intermediate BZDs	0.08	0.87	3.07	7.86	18.56	6.15
Intermediate-long BZDs	0.26	1.33	3.91	5.78	6.77	4.03
Z-drugs	0.01	0.06	0.17	0.70	1.60	0.50
Total	0.34	2.04	6.39	12.61	24.54	9.58
**Women**						
Short-intermediate BZDs	0.08	1.81	5.84	15.57	35.79	13.38
Intermediate-long BZDs	0.28	2.47	5.64	10.27	11.74	6.97
Z-drugs	0.01	0.05	0.33	1.45	2.85	1.07
Total	0.34	3.93	10.32	23.35	45.08	18.84
**All**						
Short-intermediate BZDs	0.08	1.32	4.38	11.55	28.28	9.73
Intermediate-long BZDs	0.27	1.88	4.73	7.93	9.57	5.48
Z-drugs	0.01	0.05	0.24	1.06	2.31	0.79
Total	0.34	2.95	8.25	17.76	36.13	14.16

Long-term consumption of BZDs (cumulative DDD **≥** 180) was 10% in subjects between 25 and 44 years old, 22% in those between 45 and 64 years old, and 36% in those over 65 years old. Therefore, consumption was approximately 3 times more frequent in older people than in young adults. This long-term consumption was also associated with an increase in the prevalence of insomnia (0.64% vs. 2.63%; Pearson’s chi-squared *p*-value < 0.001)).

**Fig. 1:**
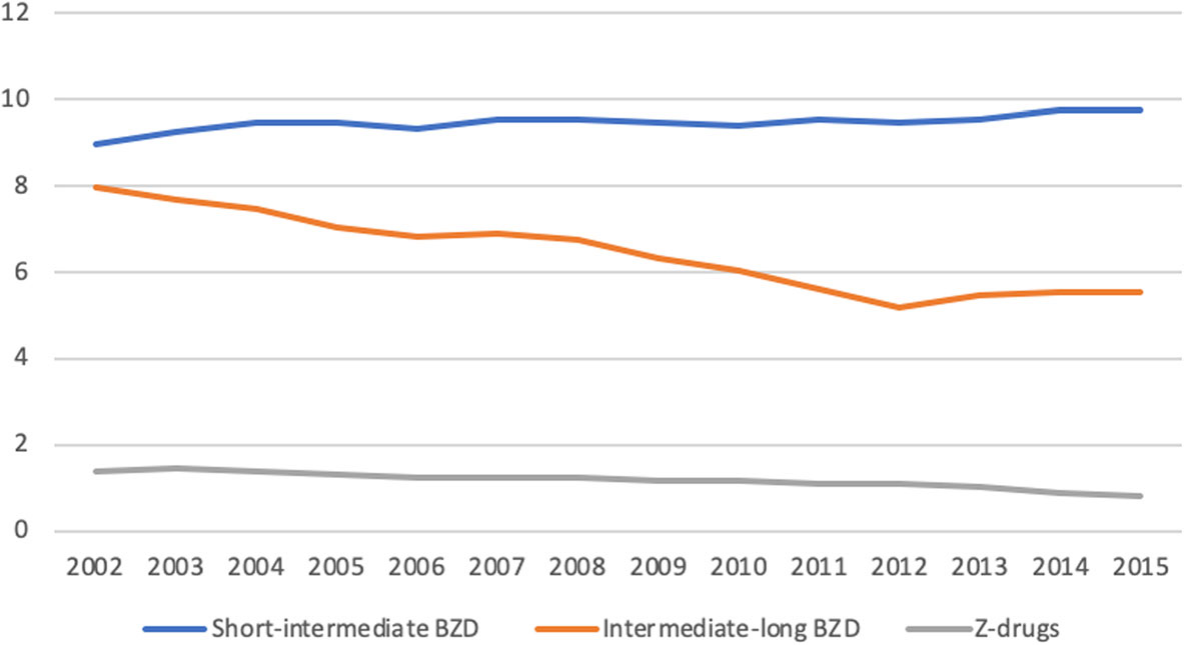
Percentage of benzodiazepine consumption by type from 2002 to 2015

If we consider the evolution of global dispensing prevalence during the study period, a slight decrease was observed, from 15.3% in 2002 to 14.2% in 2015 (Fig. 1). Regarding sex, during the study period, consumption in men decreased from 10.6 to 9.6% and in women from 19.6 to 18.8%.

**Fig. 2:**
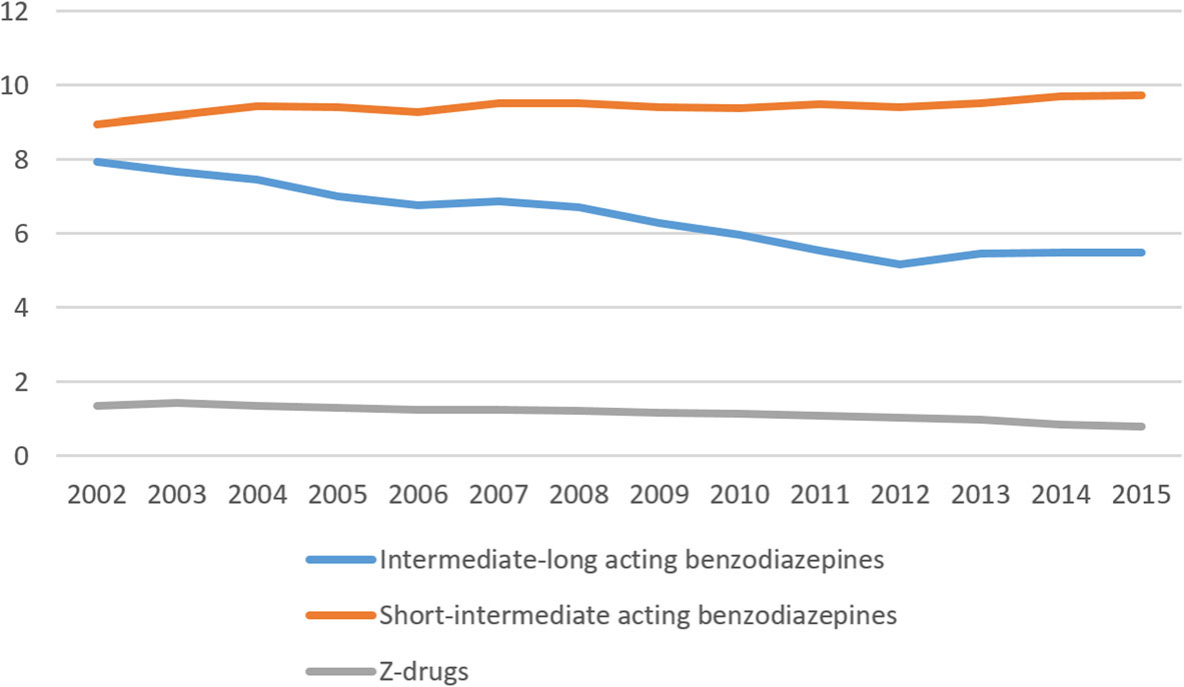
Trends of percentage of short-intermediate, intermediate-long half-life benzodiazepine and Z-drug consumption by sex from 2002 to 2015

This decrease between 2002 and 2015 was attributed to a decrease in the consumption of BZDs with intermediate-long half-lives (8.0% vs. 5.5%), as well as a decrease in the consumption of Z-drugs (1.4% vs. 0.8%). However, there was an increase in the consumption of short-intermediate half-life BZDs (8.9% vs. 9.7%), especially in women, who had an increase from 11.7 to 13.4%, while it remained constant in men (6.1 vs. 6.1%) (Fig. 2).

**Fig. 3:**
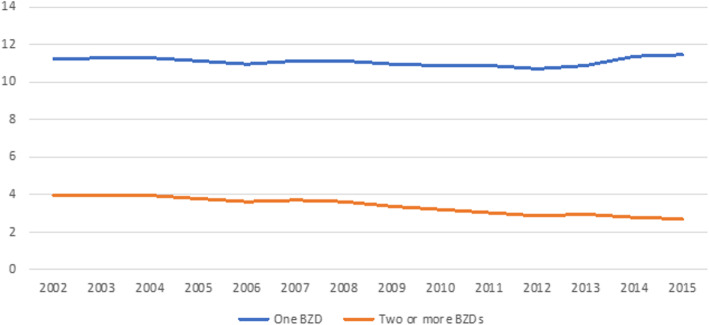
Trend of percentage of patients who are taking two or more BZDs at the same time compared with those who are taking only one BZD from 2002 to 2015

When we considered the number of BZDs that the patients were taking, we found that in 2015, 2.7% of the population consumed two or more BZDs, showing a progressive decrease from 2002, when the prevalence was 4.1% (Fig. 3). The most common BZDs used were lorazepam (36.3%), diazepam (25.1%), and lormetazepam (24.4%).

## Discussion

The results of the present study demonstrate a high prevalence in the prescription of BZDs and Z-drugs in a large population-based cohort throughout the observation period. Despite the insistence on the need to reduce the use of these drugs, there was only a slight decrease (2.47%) in the consumption of long half-life BZDs during the 13 years follow-up period, while no differences in the consumption of short half-life BZDs or Z-drugs.

According to the prescription patterns observed in Europe [2, 37–44] and US [45], the use of BZDs in this region of Catalonia was greater in women, especially elderly women.

In primary care, insomnia (42%) and anxiety (36%) are the most common indications for new prescriptions of BZDs and justify the long-term use of BZDs [46]; however, in our study, anxiety was much more frequent (24%) than insomnia (2%).

Clinical guidelines recommend that BZDs and other hypnotics should only be used in the short term in cases of severe insomnia and should only be initiated after considering non-pharmacological measures, such as sleep hygiene, stimulus control and relaxation measures [47]. However, in line with the high rates of BZD use in elderly patients, a qualitative analysis of in-depth interviews with primary care physicians from Philadephia revealed that most prescribers did not consider the continued use of BZDs in older adults to be a public health problem [48] and perceived that these drugs were more effective than non-pharmacological approaches for insomnia [49, 50]. No similar data have been published from Spanish GP doctors. In our sample, there was a disagreement between the prevalence of insomnia or anxiety and the consumption of BZDs [51, 52], which has also recently been observed in a study with a different health system model [53]. These data suggest that some health issue as insomnia or anxiety probably are not always coded in the informatic system by the GP doctors [54].

In some recent studies on the trend of BDZ prescriptions in Western countries, an increase in consumption has been observed [55, 56], while other studies have shown a stabilization or some decrease [34, 45, 57], depending on the characteristics of the population, legal aspects related to the prescription, and the period of follow-up.

When we analysed by the type of BZD used, we observed 66.9% consumption of short-intermediate half-life BZDs, in agreement with the results of a similar study conducted in the US [7]. Similarly, as observed in our study, approximately one quarter of the prescriptions of BZDs in adults were for long half-life BZDs. This proportion was consistent with a recent study of the use of BZDs among older adults in Quebec, where 24.3% of consumers received long half-life BZDs [58]. Long half-life BZDs may present risks in older people that are associated with the drug’s long half-life [59], as well as age-related changes in the pharmacokinetic and pharmacodynamic profiles of these compounds [60].

In our study, BZD consumption trends according to half-life and sex showed an increased use of short-intermediate half-life BZDs from 2002 to 2015 in women (11.7% vs. 13.4%), but use remained constant in men (6.1 vs. 6.1%). Studies conducted in northern Europe [61, 62] suggested that these prescription differences by sex were due to clinical reasons. Mendelson et al. showed that men consumed more long half-life BZDs than women; these long half-life BZDs were mainly indicated for anxiety, with insomnia being the main indication for short half-life BZDs [63].

In our observation period of 14 years, a more important reduction was observed in the use of long half-life BZDs, while consumption of the short half-life BZDs remained stable. Benzodiazepines are relatively safe for short-term use (2 to 4 weeks), but their safety has not been established beyond that period, and dependence develops in approximately half of patients who use benzodiazepines for longer than 1 month [64]. Short-intermediate half-life BZDs are typically used as hypnotic agents (e.g., triazolam), and long half-life BZDs are used as anxiolytic or anticonvulsant agents (e.g., diazepam and clonazepam). There is modest evidence that benzodiazepines with a shorter half-life are associated with a greater risk of dependence [65]. Perhaps this greater risk of dependence effect could explain the fact that there was no reduction in their consumption during the observation period. Lorazepam and alprazolam, as described in similar studies in Spain, were the most commonly used active agents among the anxiolytics [66]. Lorazepam is indicated for the treatment of anxiety associated with depression, and alprazolam is an anxiolytic with antidepressant activity.

In Ireland, the average number of DDDs of the benzodiazepines prescriptions dispensed per year decreased during the study period, while the average number of DDDs of Z drugs prescriptions dispensed per year remained largely stable over time [57]. In one Norwegian study, as many as 18% of the BZD users were high dose users, which was defined as the use of 180 DDD/year [67]. In a Danish study, the risk of exceeding a yearly dose of 180 DDD was highest among the oldest patients (eight-fold among patients older than 85 years) [68], similar to what we have observed in our study.

Despite the knowledge of most primary care physicians about the guidelines that warn against the use of long-term BZDs in the elderly population, few believe that this practice represents a serious clinical threat, and many do not feel prepared to address the problem with their patients [69]. Strategies aimed at implementing measures focused on clinical interventions that combine clinical education and drugs review [70] and re-evaluation of the benefits and risks associated with the use of BZDs can reduce their long-term prescription in primary care [71].

According to the data about Z drug prescription and comparisons to other population database studies, the observed prescribing rates for Z-drugs were lower than those in most recently published data from various countries across Europe [72–74], Asia [75] and the US [56]. It is difficult to make direct comparisons between the observed prescribing rates and those reported in other countries because of differences in the nature of the datasets, sample populations and time periods examined [76–78].

### Limitations

This study had a series of limitations. First, the dispensing data from the Public Health System included the drugs that were obtained by the population under study, not the actual use of the drug. Second, there were no data available on the specific clinical indications or actual suitability of BZD use in our study population. Third, the actual consumption of these drugs could have been greater than that reflected in this study, since private dispensers were excluded and the denominator considered was the population census of the Health Region of Lleida, a population slightly larger than that obtaining drug under the Public Health System [52]. Finally, the estimation of consumption through the DDD value established by the WHO has additional limitations, since sometimes there may be differences between the DDD value established by the WHO and the actual dose used in clinical practice. However, this technical unit of measurement allows the comparison of consumption data between different countries. Finally, although the population included in the study was representative of the general population, it is not possible to ensure that the prescribing habits of family physicians were representative of the national prescribing habits of all general practitioners.

## Conclusions

This study provides a description of trends regarding BZDs and Z-drugs use over a thirteen years period. The consumption of these drugs has been stable despite concerns about their safety and remains especially high in the elderly population, who are most sensitive to the possible side effects of these drugs. While consumption of long half-life BZDS and Z-drugs has decreased, the consumption of short half-life BZDs has remained stable. In addition, this study also shows a concerning tendency to co-consume these drugs. Given that there are treatment alternatives with fewer side effects for many of the indications for which these drugs are used, especially for the elderly, efforts should be made to better educate physicians and patients regarding these alternatives and reduce the inappropriate long-term use of BZDs and Z-drugs.

## Data Availability

The datasets used and/or analysed during the current study are available from the corresponding author on reasonable request.

## Abbreviations

BZDs: Benzodiazepines
Z-drugs: Drugs related to benzodiazepines
ATC: Anatomical therapeutic classification
DDD: Defined daily dose
WHO: World Health Organization
ICD-9-CM: International Classification of Diseases, 10th revision (2016), Clinical Modification
